# Invasive mold infection in pediatric acute lymphoblastic leukemia—a detailed analysis of the clinical trial AIEOP-BFM ALL2009

**DOI:** 10.1038/s41375-026-03084-0

**Published:** 2026-07-29

**Authors:** Thomas Lehrnbecher, Simone Cesaro, Julia Alten, Andishe Attarbaschi, Draga Barbaric, Konrad Bochennek, Nicole Bodmer, Valentino Conter, Sarah Elitzur, Anja Möricke, Martin Schrappe, Jan Stary, Ester Zapotocka, Martin Zimmermann, Andreas H. Groll

**Affiliations:** 1 Divison of Pediatric Hematology and Oncology, Department Hospital of Pediatrics, Johann Wolfgang Goethe-University, Frankfurt, Germany; 2 Paediatric Haematology Oncology, Department of Mother and Child, Azienda Ospedaliera Universitaria Integrata, Verona, Italy; 3 Pediatric Hematology-Oncology, University Hospital Schleswig-Holstein, Campus Kiel, Kiel, Germany; 4 St. Anna Children’s Hospital, Medical University of Vienna, Vienna, Austria; 5 St. Anna Children’s Cancer Research Institute, Vienna, Austria; 6 Kids Cancer Centre, Sydney Children’s Hospital, Randwick 2031, NSW 2031 Australia; 7 School of Clinical Medicine, UNSW Sydney, NSW, Australia; 8 University Children’s Hospital Zurich, Zurich, Switzerland; 9 Tettamanti Center, Fondazione IRCCS San Gerardo dei Tintori, Monza, Italy; 10 Schneider Children’s Medical Center and Gray Faculty of Medical and Health Sciences, Tel Aviv University, Tel Aviv, Israel; 11 Department of Oncology, St. Jude Children’s Research Hospital, Memphis, TN USA; 12 Czech Working Group for Pediatric Hematology, Prague, Czechia; 13 Department of Pediatric Hematology/Oncology, University Hospital Motol and Homolka, 2nd Faculty of Medicine, Charles University, Prague, Czechia; 14 Department of Pediatric Hematology/Oncology, Hannover Medical School, Hannover, Germany; 15 Infectious Disease Research Program, Department of Pediatric Hematology and Oncology and Center for Bone Marrow Transplantation, University Children’s Hospital Münster, Münster, Germany

**Keywords:** Infectious diseases, Epidemiology

## To the Editor:

Invasive fungal disease (IFD) is an important cause of morbidity in immunocompromised children and negatively impacts overall outcome [1]. Unfortunately, detailed contemporary analyses on the incidence and clinical characteristics of mold infections, including invasive aspergillosis, mucormycosis, and with rare molds (e.g., *Fusarium* spp) in large homogenous pediatric patient populations at risk, such as patients with de novo acute lymphoblastic leukemia (ALL) as the most common malignancy in childhood, are lacking. These data, however, are important for optimizing diagnostics, prophylaxis, and therapy.

Study population, antineoplastic treatment, and data collection have already been reported in detail [1]. Briefly, a total of 6136 children with de novo ALL enrolled in the international, multi-center, and prospectively randomized trial AIEOP-BFM ALL2009 (EudraCT 2007-004270-43) were included in the analysis. The study was performed in seven countries between June 1, 2010, and February 28, 2017. The trial was approved by the national and local review boards and was conducted in accordance with the Declaration of Helsinki and national laws. Informed consent was obtained from the guardians of each patient as required by ethical standards and national guidelines.

All IFDs were considered as serious adverse events (SAEs) of special interest, and the report of data prospectively captured by the participating institution was mandatory. Two experts (TL/AHG) independently categorized SAEs in proven, probable, possible, or no IFD according to the revised and updated consensus definitions [2]. Statistical analysis was performed using SPSS 29.0 (IBM, USA). Comparisons were performed by Chi-squared or Fisher´s exact test, where applicable, with *P* values < 0.05 considered significant.

**Fig. 1:**
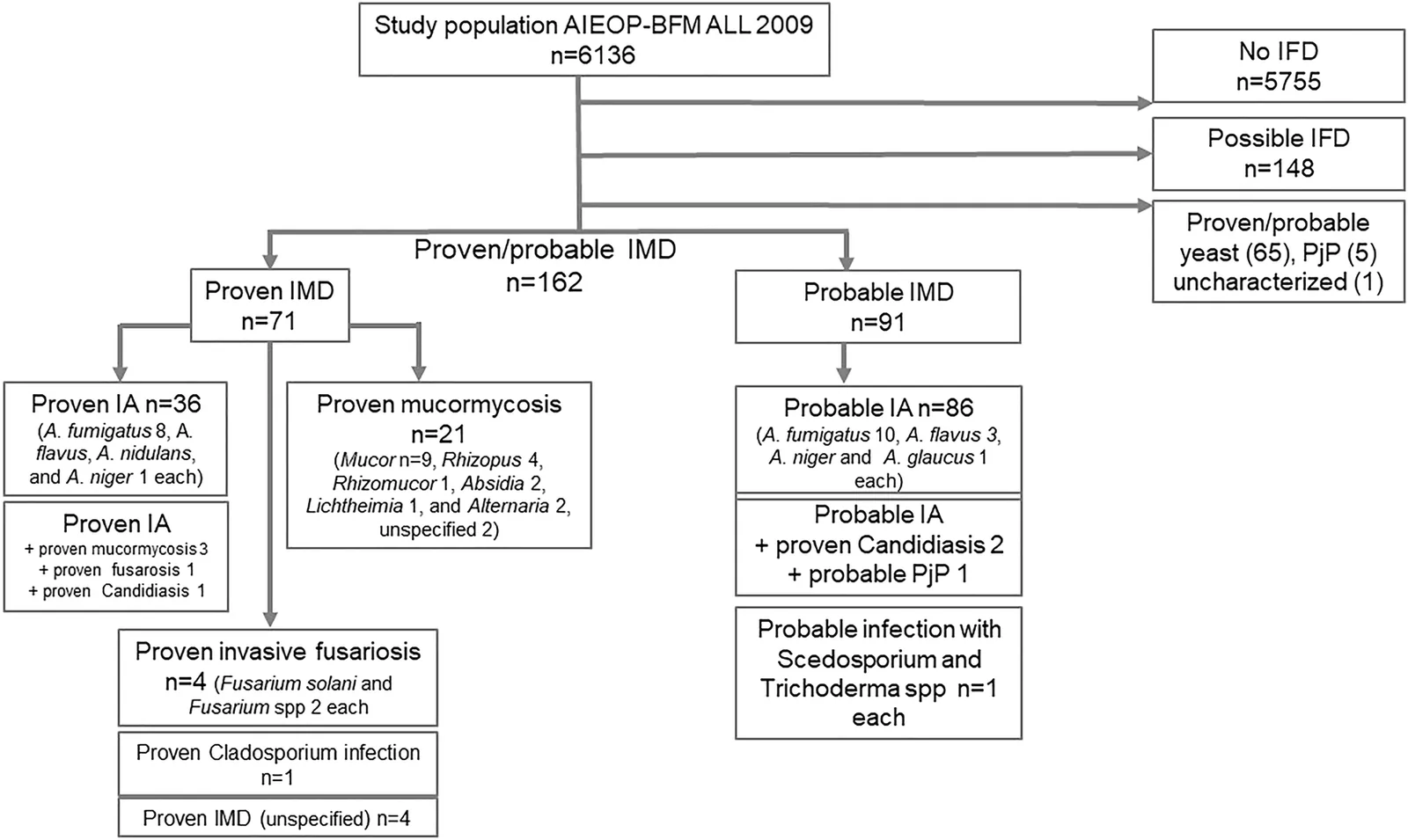
Consort diagram of patients treated according to the clinical trial AIEOP-BFM ALL2009 and analyzed for no invasive fungal disease (IFD), possible IFD, and probable and proven invasive yeast and invasive mold disease (IMD). IA invasive aspergillosis, PjP *Pneumocystis jirovecii* pneumonia.

Out of the 6136 analyzed patients, proven or probable invasive mold infection was diagnosed in 162 children [2.6%; median age (range) of the 78 girls and 84 boys 11.3 years (1.1–17.8)]. In 71 patients, invasive mold infection was proven with isolation of the following pathogens (Fig. 1): *Aspergillus* spp (*n* = 41), mucormycetes (*n* = 24), *Fusarium* spp (*n* = 5), *Cladosporium* spp (*n* = 1), and unspecified mold (*n* = 4). Probable invasive mold infection was diagnosed in 91 patients, with invasive aspergillosis in 89 patients, and with *Scedosporium prolificans* and *Trichoderma harzianum* in one patient each. Eight patients (4.9%) were diagnosed with a co-infection: in five patients, in addition to *Aspergillus* spp, mucormycetes (*n* = 3), *Fusarium* spp, and *Candida* spp (*n* = 1 each) were isolated, and two patients with probable aspergillosis had a co-infection with *Candida* spp, and one with *Pneumocystis jirovecii* (Fig. 1).

The risk of invasive mold infection was significantly higher in patients stratified as High-Risk (HR) compared to Standard (SR) or Intermedium (MR) risk leukemia [73/1403 (5.2%) versus 89/4724 (1.9%); *P* < 0.0001], older patients (median age, 11.4 versus 5.1 years, *P* < 0.0001), and in patients with T-ALL than in those with precursor-B-ALL [44/871 (5.1%) versus 117/5233 (2.2%); *P* < 0.0001]. Patients with Down Syndrome had a comparable incidence of invasive mold infection as the overall patient population [3/141 (2.1%)]. Most infections occurred in induction/consolidation therapy [93/162 (57.4%)], less often during re-intensification [45/162 (27.8%)], or the high-risk cycles [21/162 (13.0%)]. The median time (range) from the diagnosis of ALL to the invasive mold infection was 72 days (6–458). More than one-third of invasive mold infections occurred within the first 6 weeks of therapy [58 patients (35.8%); the median (interquartile range) number of days after diagnosis of ALL was 32 (27 and 36)]. The infection affected only one site in 111 (68.5%) and was disseminated in 51 (31.5%) patients, and was diagnosed in the lung and the brain in 137 (84.6%) and 30 (18.6%) of the patients, respectively. 1-year mortality of invasive mold infection was 14.2%, and all but one patient died within the first 6 weeks after the infectious event.

**Fig. 2:**
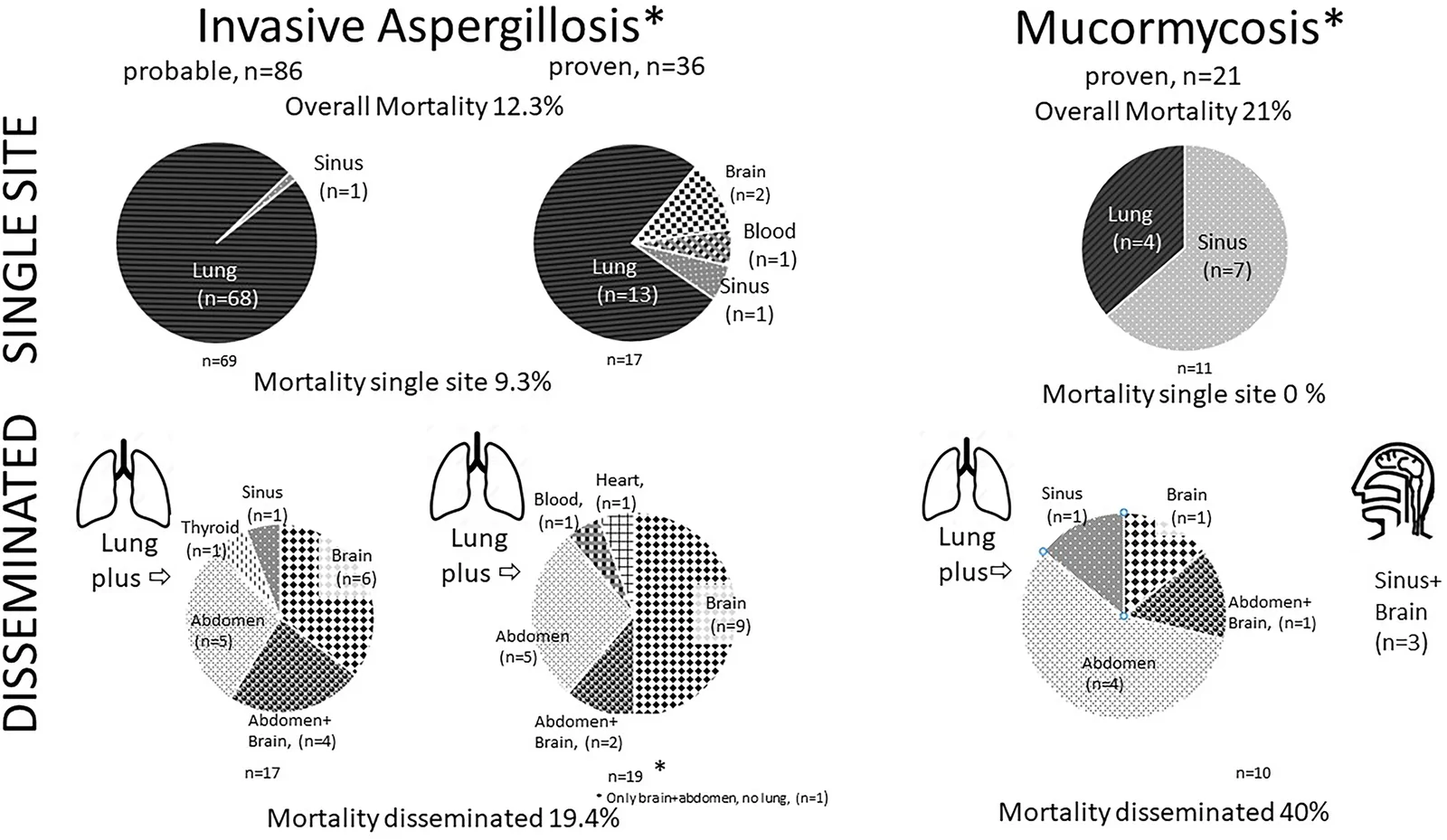
Affected sites and mortality rates of probable and proven invasive aspergillosis and mucormycosis. *Without co-infection.

For a detailed analysis of invasive aspergillosis, the eight patients with co-infection were not included (five in proven, three in probable aspergillosis). The pathogen could be characterized in 11 patients (Fig. 1). In the 122 analyzed patients with proven/probable invasive aspergillosis, the disease was limited to one organ or disseminated in 86 (70.5%) and 36 (29.5%) patients, respectively, affecting the lung in 116 (95.1%) and the central nervous system (CNS) in 24 (19.7%) patients (Fig. 2). Other organs such as sinus, thyroid, heart, abdominal organs and blood were less often affected (Fig. 2). The overall 1-year mortality of invasive aspergillosis was 12.3% (15/122), with 13.9% in proven (5/36) and 11.6% (10/86) in probable infection. There was a trend that disseminated disease had poorer outcome than localized disease (overall: 7/36 versus 8/86; proven: 3/19 versus 2/17; probable: 4/17 versus 6/69), although the difference did not reach statistical significance (Fig. 2).

Three out of the 24 patients in whom mucorales had been identified had a co-infection with *Aspergillus* spp and were not considered for the detailed analysis. In the remaining 21 patients, *Mucor* (*n* = 9), *Rhizopus* (4), Rhizomucor (1), *Absidia* (2), *Lichtheimia* (1), and Alternaria (2) were identified, whereas the mucorales could not be specified in 2 patients (Fig. 1). The disease was limited to one organ in 11 (52.4%) patients [sinus (*n* = 7), lungs (*n* = 4)], and disseminated in ten patients (47.6%) (Fig. 2). Overall, the sinus was affected in 11 patients (52.4%), the lungs in 10 patients (47.6%), and the CNS in 5 patients (23.8%). A total of four patients (19%) died of invasive mucormycosis, all with disseminated infection.

Five patients suffered from fusariosis, one of them had a co-infection with *Aspergillus* spp. Three of the four patients without co-infection were stratified as HR-patients. The infection occurred at a median (range) of 173 days (32–190) after diagnosis of ALL. Three patients had disseminated disease (skin/lung; skin/blood; blood), whereas in one patient, the infection affected the skin only. Three patients survived, whereas the patients with fusariosis of the blood died within 1 day of infection.

Our analysis of a large population of children treated for ALL demonstrated (1) an incidence of proven and probable invasive mold infection of 2.6%, (2) that the fungal infection occurred relatively early during therapy and significantly more often affected older and HR- than MR/SR patients, which might have an impact on strategies of antifungal prophylaxis, and (3) that the overall mortality was 13.5%, all of which corroborate results of recent studies [3–5]. Similar to analyses evaluating pediatric patients with a variety of underlying malignancies, we observed in our study a preponderance of infections with molds versus yeast as well as of infections with *Aspergillus* versus non-*Aspergillus* molds [3, 6–8], but IFD epidemiology is known to differ geographically. Importantly, we found 4.9% of patients had a co-infection of *Aspergillus* with other molds or yeast, which might have important implications for the choice of therapy.

Dissemination of aspergillosis and mucormycosis was seen in up to half of the patients. Corroborating reports of children with various cancers [8], the lung was the primary organ of infection in aspergillosis (116/122) and was significantly more often affected than in mucormycosis (10/21; *P* < 0.00001). In contrast, sinus involvement was seen significantly more often in mucormycosis compared to aspergillosis (11/21 versus 3/122; *P* < 0.00001). Importantly, in our analysis, in both invasive aspergillosis and mucormycosis, the CNS was affected in almost 20% of the children. Data on CNS-IFD are mostly based on retrospective case series only and, unfortunately, cannot provide any information on the incidence of the affection of the brain [9,10], which underlines the importance of our findings. Compared to our findings, previous studies evaluating mixed populations of children with leukemia reported lower incidences of CNS-IFD [3, 5], which might be explained by the retrospective nature of these studies or an incomplete diagnostic work-up. In that respect, current pediatric-specific guidelines recommend cranial imaging in children with cancer in whom proven or probable pulmonary mold infection was diagnosed [11]. In our study, the skin was only affected in patients with fusariosis, but not in those with aspergillosis or mucormycosis, which is in contrast to the high incidence of skin involvement reported in other studies [8]. The difference might be explained by a low incidence of *A. flavus*, which has been associated with cutaneous infection [12].

In our study, mortality rates for invasive aspergillosis and mucormycosis were comparable to those reported in various pediatric malignancies, and indicate that outcome has improved over the last decades [13–15]. Notably, compared to invasive aspergillosis, mortality in mucormycosis was higher, and disseminated disease had a poorer outcome than localized infection, although the differences were statistically not significant, which might be due to the overall low numbers.

The strength of our analysis is the fact that we evaluated an extremely large population of children with de novo ALL enrolled in a multi-center international randomized controlled clinical trial. This provides a solid denominator, which is not available in retrospective analyses of children with various malignancies. In addition, the data quality is extremely high due to the fact that reporting of the prospectively collected SAEs was mandatory and systematically monitored. However, we recognize that our study also has some important limitations. First, we do not have data on antifungal prophylaxis, which impacts risk and epidemiology of invasive mold infection. Second, diagnostic strategies might have differed across the centers and have influenced the results, as these tools were used at the discretion of the treating physician.

Our data are unique and will help to optimize antifungal prophylaxis and diagnostic approaches in children with ALL, and hopefully will ultimately help to improve outcomes in this patient population.

## Data Availability

Under the permission that national data protection requirements are fully met, access to individual data may be made available upon reasonable request to the authors.
